# Usefulness of AI-Equipped Endoscopy for Detecting Colorectal Adenoma during Colonoscopy Screening: Confirm That Colon Neoplasm Finely Can Be Identified by AI without Overlooking Study (Confidential Study)

**DOI:** 10.3390/jcm12196332

**Published:** 2023-10-02

**Authors:** Kazuhiro Mizukami, Erina Fushimi, Ryota Sagami, Takashi Abe, Takao Sato, Shohei Terashi, Masahide Fukuda, Hidefumi Nishikiori, Takayuki Nagai, Masaaki Kodama, Kazunari Murakami

**Affiliations:** 1 Department of Gastroenterology, Faculty of Medicine, Oita University, 1-1, Idaigaoka, Hasama, Yufu, Oita 879-5593, Japan; 2 Department of Gastroenterology, Oita San-ai Medical Center, 1213 Ichi, Oita 870-1151, Japan; 3 Department of Gastroenterology, Oita Koseiren Tsurumi Hospital, 4333, Tsurumi, Beppu, Oita 874-8585, Japan; 4 Department of Advanced Medical Sciences, Faculty of Medicine, Oita University, 1-1, Idaigaoka, Hasama, Yufu, Oita 879-5593, Japan

**Keywords:** colonoscopy, artificial intelligence, colon adenoma, adenoma detection rate, screening

## Abstract

In the present prospective case series study, we investigated the lesion-detection ability of an AI-equipped colonoscopy as an addition to colonoscopy (CS) screening. Participants were 100 patients aged ≥20 years who had not undergone CS at the study site in the last 3 years and passed the exclusion criteria. CS procedures were conducted using conventional white light imaging and computer-aided detection (CADe). Adenoma detection rate (ADR; number of individuals with at least one adenoma detected) was compared between the conventional group and the CADe group. Of the 170 lesions identified, the ADR of the CADe group was significantly higher than the ADR of the conventional group (69% vs. 61%, *p* = 0.008). For the expert endoscopists, although ADR did not differ significantly, the mean number of detected adenomas per procedure (MAP) was significantly higher in the CADe group than in the conventional group (1.7 vs. 1.45, *p* = 0.034). For non-expert endoscopists, ADR and MAP were significantly higher in the CADe group than in the conventional group (ADR 69.5% vs. 56.6%, *p* = 0.016; MAP 1.66 vs. 1.11, *p* < 0.001). These results indicate that the CADe function in CS screening has a positive effect on adenoma detection, especially for non-experts.

## 1. Introduction

Colorectal cancer (CRC) remains one of the most common cancers with high morbidity and mortality rates in the world, although it is gradually decreasing due to widespread screening [1]. Studies have shown that endoscopic resection of neoplastic lesions by colonoscopy (CS) reduces the morbidity and mortality of CRC [2,3,4], and CS screening tests are emphasized in guidelines around the world to reduce CRC mortality [5,6]. A large cohort study conducted in the United States showed an approximately 70% reduction in deaths due to CRC after screening colonoscopy was introduced [7]. Zauber et al. also showed in the National Polyp Study, a randomized controlled trial conducted in the United States, that removal of all tumor polyps in the colon can reduce CRC deaths by approximately 50% [4].

On the other hand, it is also true that a certain percentage of CRCs occur after routine CS, and these are called post-colonoscopy colorectal cancers (PCCRCs). Anderson R et al. reported that the development of 73% of PCCRCs was determined to be affected by technical endoscopic factors, 17% of PCCRCs by administrative factors (follow-up procedures delayed/not booked by administrative staff), and 27% of PCCRCs by decision-making factors [8]. Many reports also tell us that PCCRCs are associated with the rate of colorectal tumors missed by CS [8,9,10]. In a meta-analysis of 43 publications and more than 15,000 tandem CSs, the missed rate was 26% for adenomas, 9% for advanced adenomas, and 27% for serrated polyps [10]. The major quality indicators for tumor identification in CS are said to be the ileal reach rate [2,11,12], bowel cleansing rate [2,13], and adenoma detection rate (ADR) [2,14,15]. While the ileal reach rate and bowel cleansing rate are inescapable in part due to problems with the patient’s own colon structure and bowel peristalsis ability, ADR is largely dependent on the endoscopist’s skill. Kaminski et al. reported that the risk of PCCRC was more than 10 times higher for endoscopists with an ADR of less than 20% compared to endoscopists with an ADR of 20% or higher [14].

Regarding the search for CRC by endoscopy, improvements such as image-enhanced endoscopy have been made to increase the visibility of tumors [16]. Atkinson et al. showed that, in a meta-analysis of randomized, controlled trials, narrow-band imaging (NBI) resulted in a higher ADR than conventional white light imaging (WLI) [17]. Linked color imaging (LCI) is considered the image-enhanced endoscopy with the best visibility of colorectal lesions among LCI, blue light imaging (BLI), and WLI [18]. LCI rearranges the color information obtained with narrow-band lights and white light to emphasize the tonal difference between reddish and faded tones. Shinozaki et al. showed in a meta-analysis that the use of LCI improved the detection of polyps and adenomas and of previously missed polyps compared with WLI [19].

In recent years, endoscopic devices equipped with artificial intelligence (AI) have appeared on the market and attracted much attention. The current AI installed in a colonoscope can automatically recognize subtle lesions in real time and even predict the histology of the lesions [20]; it can be expected to improve the ADR in actual clinical practice and change CS screening to a more flawless examination in the future.

In this study, a colonoscope equipped with an AI function was used to verify how much lesion detection by AI adds to the usual CS screening. In addition, we examined under what situation AI could benefit lesion detection.

## 2. Materials and Methods

### 2.1. Participating Institutes and Patients

This study was conducted as a prospective case series study from September 2021 to June 2022 at three endoscopy units including a university hospital in Oita prefecture. All hospitals are high-volume centers with at least two expert endoscopists. This study was performed in compliance with the Declaration of Helsinki and the ethical guidelines for medical and health research involving human subjects, and it was conducted after the ethics committees of all three institutes approved the protocol and informed consent form. This study was registered with the University Hospital Medical Information Network (UMIN No. 000045456).

Patients aged 20 years or older who had not undergone CS at the study site for at least 3 years were enrolled. The following patients were excluded: (1) patients undergoing emergency endoscopy; (2) patients with a history of intestinal resection or severe intestinal adhesions; (3) patients with chronic bowel disease such as inflammatory bowel disease; (4) patients whose preparation was unsatisfactory because the same area is observed twice in this study, so if the preparation is inadequate, the first time will be overwhelmingly disadvantageous due to cleaning, etc.; and (5) patients who were judged inappropriate by the doctor in charge of this study in order to ensure patient safety that could not be foreseen during the study planning phase. In addition, after enrollment, patients in whom the cecum was not intubated during CS, patients who scored “poor” or “inadequate” on the Aronchick Scale [21] in bowel preparation, and patients who scored ≤5 points on the Boston Bowel Preparation Scale [22] were also excluded. Written, informed consent was provided by all patients before enrollment.

### 2.2. Endoscopic Equipment and AI System

The LASEREO system (light source LL-7000/BL-7000, processor VP-7000, Fujifilm Corp., Tokyo, Japan) and a high-definition monitor were used, and all procedures were performed with a high-resolution video colonoscope (EC-L600ZW7/760ZP Fujifilm Corp., Tokyo, Japan). The CADEYE was used as the AI system (Fujifilm Corp., Tokyo, Japan). The CADEYE is an AI system for the detection and characterization of colorectal polyps during CS. The CADEYE can automatically detect the location of a suspected polyp, showing a colored box around it and emitting a detection sound, in WLI and LCI modes (computer-aided detection, CADe). Then, the CADEYE can differentiate between neoplastic and hyperplastic lesions when the operator switches to BLI mode (computer-aided diagnosis; CADx) [20].

### 2.3. Study design

All endoscopists inserted the fiber into the cecum in WLI mode without CADe, and then, they washed the colon as much as possible before starting observation to ensure optimal visualization.

**Figure 1:**
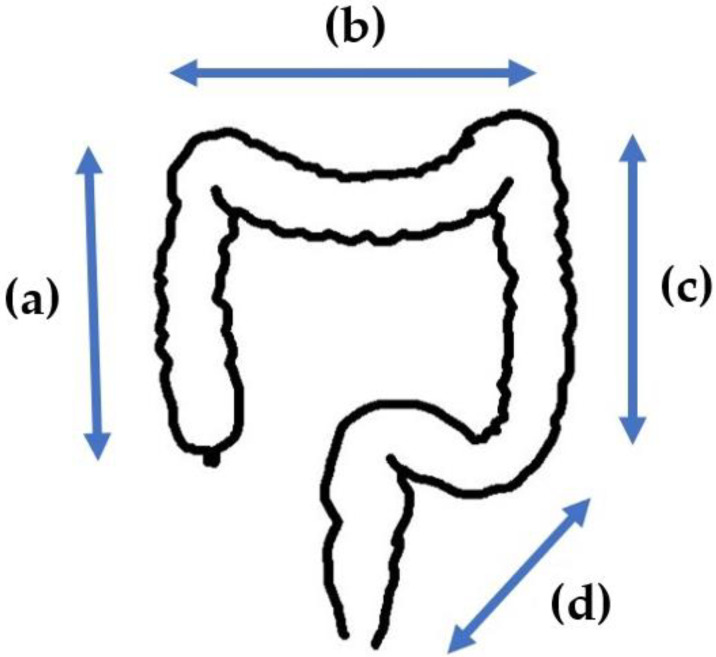
CS procedure in this study. First, observation was performed from the cecum to the ascending colon (**a**) while withdrawing the colonoscope in WLI mode without CADe. Then, returning to the cecum, the same area was observed again using CADe. After that, the transverse colon segment (**b**), the descending colon segment (**c**), and the sigmoid colon to rectum segment (**d**) were observed using the same procedure, with conventional WLI followed by observation with CADe.

The CS observation procedure in this study is shown in Figure 1. First, observation was performed from the cecum to the ascending colon (a) while withdrawing the colonoscope in WLI mode without CADe. Then, returning to the cecum, the same area was observed again using CADe. After that, the transverse colon segment (b), the descending colon segment (c), and the sigmoid colon to rectum segment (d) were observed using the same procedure, with conventional WLI followed by observation with CADe.

Insertion time was the time from colonoscope insertion into the anus to arrival at the cecum, and observation time was calculated for each mode by recording the start and end time of observation in each segment.

Target lesions were defined as precancerous lesions (adenoma, dysplasia, serrated lesions) and carcinoma according to the World Health Organization guidelines [23] and as those judged “neoplastic” by either naked eye observation by the endoscopist or the diagnostic mode of CADEYE. When the lesions were determined to be target lesions, hot/cold biopsy, hot/cold snare polypectomy, and endoscopic mucosal resection (EMR) were performed on all lesions after the completion of the two types of observation, and histological diagnosis was performed. When endoscopic submucosal resection (ESD) or surgical operation was indicated, the final histological diagnosis was carried out using resected specimens. Features such as location, size, and shape were recorded for all lesions detected during CS.

### 2.4. Endoscopists

A total of 12 endoscopists were in charge of the examinations (4 experts, 8 non-experts). An expert was defined as an experienced endoscopist who was a Board-Certified Trainer and Councilor of The Japan Gastroenterological Endoscopy Society. The specialists had a median of 19 (13–21) years of experience in CS, performing a mean of 6000 (3500–20,000) CS cases. The non-experts had a median of 4 (1–11) years of experience in CS, performing a mean of 400 (150–5000) CS cases.

### 2.5. Evaluation

The primary endpoint was the ADR (proportion of individuals undergoing a complete CS who had at least one adenoma detected) compared between the conventional group (observation without CADe) and the CADe group (observation with CADe). The secondary endpoint was the mean number of detected adenomas per procedure (MAP) compared between the conventional group and the CADe group. In addition, the ADR and MAP of the conventional group and the CADe group were compared in the expert and non-expert groups. We also selected cases with multiple adenomas and examined the percentage of these cases in which endoscopists were able to detect all adenomas that CADe could detect.

Adverse events were also recorded to ensure the safety of this study.

### 2.6. Statistical Analysis

Data are expressed as medians (interquartile range, range). All statistical analyses were performed with EZR (Saitama Medical Center, Jichi Medical University, Saitama, Japan), which is a graphical user interface for R (The R Foundation for Statistical Computing, Vienna, Austria). More precisely, it is a modified version of R commander designed to add statistical functions used frequently in biostatistics.

For categorical variables, McNemar’s test was used when paired, and Fisher’s exact test was used when unpaired. For continuous variables, the Wilcoxon signed-rank test was used when paired, and the Mann–Whitney U test was used unpaired. Values of *p* < 0.05 were considered significant.

## 3. Results

### 3.1. Baseline Characteristics

A total of 100 cases were enrolled in this study. In no patient was the end of the ileum not reached, and none had inadequate bowel preparation, both of which were defined as exclusion criteria. Patients’ baseline characteristics are given in Table 1. The median age of the patients was 66.5 years, 51 were male, 53 were undergoing CS for the first time, 47 had not undergone CS for more than 3 years, and 30 patients were obese with BMI > 25 kg/m^2^.

### 3.2. Colorectal Observation Time

The median colorectal observation time was 416.5 (320.75, 201–1427) seconds for the conventional group and 442.5 (326.25, 207–1122) seconds for the CADe group, with no significant difference (*p* = 0.337).

### 3.3. Characteristics of Identified Lesions

A total of 170 lesions were identified, and their characteristics are given in Table 2. The location of lesions was 46 in the ascending colon, 36 in the transverse colon, 26 in the descending colon, 53 in the sigmoid colon, and 9 in the rectum, with more in the ascending colon in the expert group and more in the descending colon in the non-expert group (*p* = 0.003). Regarding lesion size, 81.8% of the lesions were less than 10 mm in diameter, with more being 5–10 mm in the expert group and more being greater than 10 mm in the non-expert group (*p* = 0.037). Histological procedures included 2 biopsies, 111 CSPs, 56 EMRs, and 1 polypectomy. The pathological diagnosis was tubular adenoma in 158 cases, tubulovillous adenoma in 7 cases, serrated adenoma in 1 case, and adenocarcinoma in 4 cases.

### 3.4. Primary Endpoint

**Figure 2:**
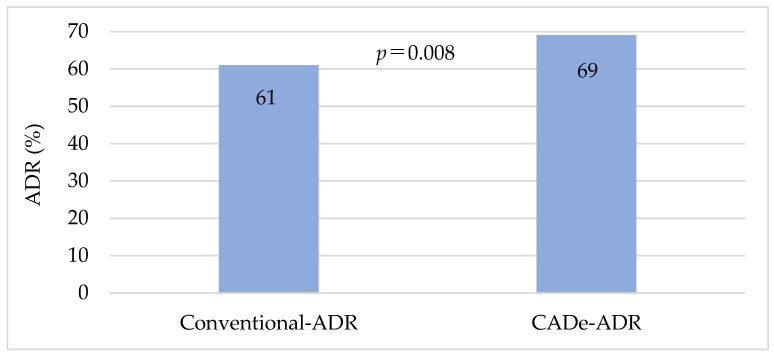
Comparison of overall conventional-ADR and CADe-ADR. Conventional-ADR: adenoma detection rate in the conventional group; CADe-ADR: adenoma detection rate using computer-aided detection. *p*-values were calculated by McNemar’s test.

The comparison of the ADR between the conventional group and the CADe group is shown in Figure 2. The ADR was significantly higher in the CADe group (CADe-ADR, 69%) than in the conventional group (conventional-ADR, 61%) (*p* = 0.008).

### 3.5. Secondary Endpoint

Of the CSs, 47 were performed by experts and 53 by non-experts. There were no significant differences in patients’ background characteristics between the expert group and the non-expert group (Table 1). The median insertion time was 265 s for the expert group and 399 s for the non-expert group, with the expert group having a significantly shorter insertion time (overall median 343.5 s) (*p* = 0.002) (Table 1). The median observation time was 379 (312, 201–682) seconds for the conventional group and 410 (307.5, 207–752) seconds for the CADe group in the expert group, whereas the median observation time was 473 (355, 247–1427) seconds for the conventional group and 508 (349, 219–1122) seconds for the CADe group in the non-expert group. There was no significant difference in either group (the expert group *p* = 0.354, the non-expert group *p* = 0.056).

A comparison of ADR and MAP between the conventional group and the CADe group by endoscopist experience is given in Table 3. In the expert group, the comparison between the conventional group and the CADe group showed no significant difference, with the conventional-ADR of 66% vs. the CADe-ADR of 68.1%. On the other hand, in the non-expert group, the conventional-ADR (56.6%) was significantly lower than the CADe-ADR (69.5%) (*p* = 0.016).

In the expert group, the MAP was 1.45 for the conventional group and 1.7 for the CADe group, and in the non-expert group, the MAP was 1.11 for the conventional group and 1.66 for the CADe group, both showing that the CADe group was significantly more likely to identify polyps (the expert group *p* = 0.034, the non-expert group *p* < 0.001).

**Figure 3:**
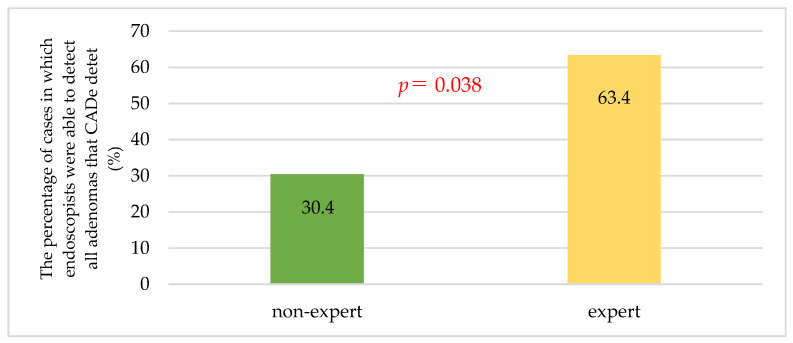
The percentage of cases in which endoscopists were able to detect all adenomas that CADe could detect. The expert group had a higher percentage of cases in which all polyps noted by CADe were detected than the non-expert group (*p* = 0.038). *p*-values were calculated by Fisher’s exact test.

Multiple neoplastic lesions were found in 45 cases (22 in the expert group and 23 in the non-expert group). Endoscopists were able to identify all adenomas noted by CADe in 63.4% of cases in the expert group and 30.4% in the non-expert group (*p* = 0.038) (Figure 3).

No adverse events, including bleeding, occurred in this study.

## 4. Discussion

One of the most significant aspects of CS screening is the detection and elimination of CRC or adenoma, which is the pre-CRC stage. On the other hand, missed lesions in CS screening are an important challenge, because they may connect directly to cancer death. Although this challenge is influenced by the conditions and environment of the CS, it is also largely dependent on the endoscopist’s observational skills. The present study demonstrates that, in CS screening, in any endoscopist, who is an expert or non-expert, the CADe function has an additive effect on the ADR, a quality indicator of CS.

Although methods to automatically detect polyps using edge detection, texture analysis, and energy maps have been studied for CADe since the 2000s, the detection rate was not stable, and real-time diagnosis was difficult [24]. However, with the advent of deep learning using graphics processing units, AI can learn a large number of images iteratively and thus understand and classify image features on its own, dramatically improving the detection rate and accuracy of lesions [25]. Currently, with the incorporation of a Convolution Neural Network, CADe has reached the point where it can detect polyps with high accuracy in real time [25].

In the field of gastrointestinal endoscopy, CADe for CS is the most investigated area. In the CS field, endoscopes with CADe have already been applied clinically, and several clinical trials with CADe have already been reported from countries other than Japan [26,27,28,29,30,31]. A systematic review of CS screening with CADe showed that the ADR without CADe was 19.3% (12.7–25.9%), whereas the ADR with CADe was 29.6% (22.2–37.0%), showing a higher ADR in CS with CADe (relative risk 1.52 (1.31–1.77)) [32]. In the present study, comparing CS performed by endoscopists alone to CS with CADe, the overall ADR improved from 61% to 69%. Corley et al. reported that a 1% increase in ADR reduces CRC incidence by 3% and death from CRC by 5% [15], and in this respect alone, the use of CADe can be considered clinically significant.

The present study also showed that CS combined with CADe is effective in improving the ADR in the non-expert group, even though there was no significant difference in the ADR in the expert group. In CS, the endoscopist’s skill is one of the factors that have a significant impact on the ADR. Mehrotra A et al. reported that in a retrospective study of 104,618 CSs for 201 endoscopists, gastroenterologists, female physicians, and more recently trained physicians had higher ADR [33]. Therefore, when the trainees perform the CS, the endoscopic instructors must keep monitoring their CS procedures. In the present study, CADe was considered to be an adequate complement to ADR by non-expert endoscopists, and CADe is expected to reduce the burden on endoscopic fellows and instructors. Yamaguchi D et al. used a back-to-back method to examine the usefulness of colonoscopy screening using CADe in six endoscopy trainees, and the CADe-using group had a significantly lower adenoma miss rate (25.6% vs. 38.6%, *p* = 0.033) and number of missed adenomas per patient (0.5 vs. 0.9, *p* = 0.004) compared to the non-CADe-using group [34].

On the other hand, the current study showed that CADe also benefited CS experts. Expert endoscopists were comparable to CADe in the ADR, but in the MAP, CADe was significantly higher than experts (Table 3). Furthermore, in CS of patients with multiple tumor lesions, experts alone were only able to find all lesions that CADe could identify 63.4% of the time (Figure 3). This suggests the limitation of an endoscopist finding all adenomas on his/her own, no matter how good he/she is. Recently, fellow interventions have been reported to improve ADRs [35,36,37]. Facciorusso et al. showed that, in a multicenter, randomized trial, the fellow intervention increased the number of ADRs and adenomas detected, indicating a benefit of supervised intervention in CS (ADR 44.8% in the fellow intervention group vs. 37.1% in the control group, *p* = 0.02; MAP 0.65 ± 0.3 in the fellow intervention group vs. 0.53 ± 0.2 in the control group, *p* < 0.001) [37]. These show that fellow participation could be attributed to increased recognition of small polyps by a second set of eyes. Moreover, this study also reports that a year of fellowship training clearly impacts PDR and ADR, with increased rates associated with higher levels of training [37]. CADe itself can be a very highly trained partner, so to speak. Endoscopists can expect to maintain a high ADR by performing CS with CADe as a second set of eyes.

Also, other factors that cause endoscopists to miss adenoma may include concentration and fatigue during the examination. When examined by polyp detection rate by order of lesion detected, the expert group had 81.8% of the first polyp, 87.0% of the second polyp, 91.7% of the third polyp, and 61.9% of the fourth and subsequent polyps, showing a significant difference in the detection rate of the first to third polyps and the fourth and subsequent polyps (Fisher’s exact test; *p* = 0.021, unpublished data). Although it is difficult to assess the degree of concentration and fatigue during the examination, we cannot rule out the possibility that these conditions affected the expert group in this study. Since CADe can maintain a high detection capacity at any time, it is expected to increase the detection rate of tumors in any CS, including medical check-ups.

There are several limitations to this study. First, the sample size was small. The usefulness of AI in colonoscopy has already been reported in previous reports and the sample size in this study was not sufficient. Therefore, a CS interval of at least 3 years was defined as an eligibility criterion, and keeping the ADR above 60%, we made sure to ensure a good quality exam. Second, the study was a one-arm study, which may be biased by the fact that observation with CADe was performed after conventional observation. Third is the issue of observation time. It is recommended that CS screening observations should maintain a withdrawal time of 6 min or longer to detect adenomas [38]. In the present study, there were 34 CS cases with an ADR of 6 min or less (14 by the non-expert group and 20 by the expert group). The ADR for these cases was 67.6%, which was not significantly different from the CSs of 6 min or longer, but the withdrawal times could not be aligned, despite it being a prospective study. In addition, because of differences in lesion location and characteristics between the two groups, a strict comparison between specialists and non-specialists was not possible. This problem could be solved with a large sample size, and further studies on a national scale are expected in the future.

Currently, most prospective studies using CADe are conducted in medically advanced countries. The present study suggests that CADe may be of great value in regions with less developed medical care. As CADe becomes more widely used, it is expected that large-scale, multicenter, prospective, randomized trials will be conducted in a variety of settings. In addition, the combination of IEE and CADe can be expected to further improve the detection of colorectal adenomas. In particular, as studies in combination with LCI and CADe are conducted on a large scale, we hope to see the accuracy of CS screening increase more in the future.

## 5. Conclusions

CS with CADe can be expected to increase adenoma detection in any endoscopist and in any setting. This is especially true for non-experts.

## Figures and Tables

**Table 1:** Background of CS cases.

	Total	Experts	Non-Experts	*p*-Value
Number	100	47	53	-
Age (median (IQR, range))	66.5 (59.75, 37–89)	68 (62.5, 37–89)	65 (59, 41–85)	0.578
Male	51	27	24	0.32
CS for the first time	53	24	29	0.843
Obesity (BMI > 25)	30	12	18	0.388
Insertion time (min) (median (IQR, range))	343.5 (224.75, 89–2348)	265 (212.5, 89–1042)	399 (278, 150–2348)	0.002

*p*-values were calculated by Fisher’s exact test in males, CS for the first time, and obesity. *p*-values were calculated by the Mann–Whitney U test in age and mean intubation time. CS: colonoscopy; IQR: interquartile range; BMI: body mass index.

**Table 2:** Characteristics of neoplastic lesions found in CS.

		Total	Experts	Non-Experts	*p*-Value
Lesion area		170	82	88	
	Ascending colon	46	28	18	0.003
	Transverse colon	36	17	19	
	Descending colon	26	4	22	
	Sigmoid colon	53	28	25	
	Rectum	9	5	4	
Size					
	<5 mm	77	35	42	0.037
	5–10 mm	62	37	25	
	>10 mm	31	10	21	
Form					
	Ⅰs	97	50	47	0.783
	Ⅰsp	54	24	30	
	Ⅰp	8	3	5	
	Others	11	5	6	
Procedure (histology)					
	Biopsy	2	0	2	<0.001
	CSP	111	43	68	
	EMR	56	38	18	
	Polypectomy	1	1	0	

*p*-values were calculated by Fisher’s exact test. CS: colonoscopy; CSP: cold snare polypectomy; EMR: endoscopic mucosal resection.

**Table 3:** Comparison of ADR and MAP between the conventional and CADe groups by endoscopist career.

		Conventional Group	CADe Group	*p*-Value
ADR				
	Total	61%	69%	0.013 ^a^
	Expert	66%	68.1%	1 ^a^
	Non-expert	56.6%	69.5%	0.023 ^a^
MAP				
	Total	1.29	1.7	<0.001 ^b^
	Expert	1.45	1.7	0.034 ^b^
	Non-expert	1.11	1.66	<0.001 ^b^

^a^ *p*-values were calculated by McNemar’s test. ^b^ *p*-values were calculated by the Wilcoxon signed-rank test. ADR: adenoma detection rate; MAP: mean number of detected adenomas per procedure.

## Data Availability

The data used in the current study are available from the corresponding author upon reasonable request.
